# Can bronchodilators improve exercise tolerance in COPD patients without dynamic hyperinflation? *

**DOI:** 10.1590/S1806-37132014000200003

**Published:** 2014

**Authors:** Maria Enedina Aquino Scuarcialupi, Danilo Cortozi Berton, Priscila Kessar Cordoni, Selma Denis Squassoni, Elie Fiss, José Alberto Neder

**Affiliations:** Paraíba School of Medical Sciences, João Pessoa, Brazil; Federal University of Rio Grande do Sul School of Medicine; and Pulmonologist. Porto Alegre Hospital de Clínicas, Porto Alegre, Brazil; Department of Pulmonology, ABC School of Medicine, Santo André, Brazil; Pulmonary Rehabilitation Division, Department of Pulmonology, ABC School of Medicine, Santo André, Brazil; Department of Pulmonology, ABC School of Medicine, Santo André, Brazil; Division of Respiratory and Critical Care Medicine, Queen’s University and Kingston General Hospital, Kingston, Canada; and Professor. Federal University of São Paulo, São Paulo, Brazil

**Keywords:** Pulmonary disease, chronic obstructive, Bronchodilator agents, Exercise test, Exercise tolerance, Inspiratory capacity

## Abstract

**OBJECTIVE:**

To investigate the modulatory effects that dynamic hyperinflation (DH), defined as a reduction in inspiratory capacity (IC), has on exercise tolerance after bronchodilator in patients with COPD.

**METHODS:**

An experimental, randomized study involving 30 COPD patients without severe hypoxemia. At baseline, the patients underwent clinical assessment, spirometry, and incremental cardiopulmonary exercise testing (CPET). On two subsequent visits, the patients were randomized to receive a combination of inhaled fenoterol/ipratropium or placebo. All patients then underwent spirometry and submaximal CPET at constant speed up to the limit of tolerance (Tlim). The patients who showed ΔIC(peak-rest) < 0 were considered to present with DH (DH+).

**RESULTS:**

In this sample, 21 patients (70%) had DH. The DH+ patients had higher airflow obstruction and lower Tlim than did the patients without DH (DH-). Despite equivalent improvement in FEV_1_ after bronchodilator, the DH- group showed higher ΔIC(bronchodilator-placebo) at rest in relation to the DH+ group (p < 0.05). However, this was not found in relation to ΔIC at peak exercise between DH+ and DH- groups (0.19 ± 0.17 L vs. 0.17 ± 0.15 L, p > 0.05). In addition, both groups showed similar improvements in Tlim after bronchodilator (median [interquartile range]: 22% [3-60%] vs. 10% [3-53%]; p > 0.05).

**CONCLUSIONS:**

Improvement in TLim was associated with an increase in IC at rest after bronchodilator in HD- patients with COPD. However, even without that improvement, COPD patients can present with greater exercise tolerance after bronchodilator provided that they develop DH during exercise.

## Introduction

Lung hyperinflation is a crucial mechanism of dyspnea on exertion in COPD patients.^(^ ^1^ ^-^ ^3^ ^)^ Bronchodilator therapy can reduce static and dynamic lung volumes during exercise, increasing exercise tolerance in such patients.^(^ ^4^ ^,^ ^5^ ^)^

The current concept of the mechanisms whereby bronchodilators can improve exercise tolerance in patients with COPD focuses on the ability to reduce the rate of increase in end-expiratory lung volume (EELV) as the exercise progresses, i.e., a reduction in dynamic hyperinflation (DH). ^(^ ^1^ ^,^ ^6^ ^)^ In practice, DH can be estimated by serial measurements of inspiratory capacity (IC),^(^ ^6^ ^-^ ^8^ ^)^ which reflects EELV, given that TLC does not change significantly with exercise.^(^ ^9^ ^)^ An alternative (or complementary) mechanism of action of bronchodilators is reduction in operating lung volumes at rest, i.e., pre-exercise deflation.^(^ ^10^ ^,^ ^11^ ^)^ In this case, patients can benefit from bronchodilator use even in the absence of DH, given that there are "volume reserves" to be consumed during exercise. In any event, with the use of a bronchodilator, all patients can achieve the same EELV at peak exercise, albeit by different mechanisms (i.e., either by a reduced rate of DH or by reduced static hyperinflation).

Our objective was to investigate whether the administration of a bronchodilator results in improvement in exercise capacity in patients with moderate to severe COPD, despite the fact that bronchodilators act predominantly on exercise-related static hyperinflation or DH. The confirmation of this hypothesis would support the notion that measurements of lung hyperinflation at rest and during exercise are complementary in the evaluation of the effects of bronchodilators on exercise tolerance in such patients.

## Methods

We studied a convenience sample of 30 patients diagnosed with COPD in accordance with the Global Initiative for Chronic Obstructive Lung Disease criteria.^(^ ^12^ ^)^ The patients were over 40 years of age and had a post-bronchodilator FEV_1_ < 70% of predicted, an FEV_1_/FVC ratio < 70%, and a smoking history of more than 20 pack-years. Patients were recruited from among those treated at the COPD outpatient clinic or pulmonary rehabilitation center of our institution. The exclusion criteria were as follows: severe resting hypoxemia (SpO_2_ < 90%); comorbidities contributing to dyspnea and exercise limitation; COPD exacerbation or respiratory infection in the previous month; and contraindication to clinical exercise testing. The study project was approved by the local research ethics committee. All participants gave written informed consent.

**Figure 1:**
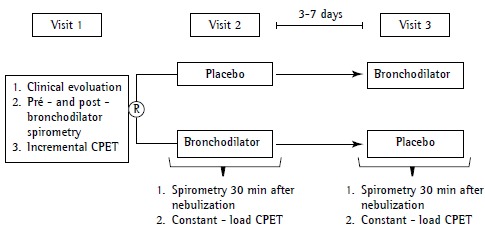
Study design. BD: bronchodilator; CPET: cardiopulmonary exercise testing; and R: randomization.

At the initial visit, all of the patients who remained eligible after their clinical and functional characteristics had been determined by spirometry performed before and after the administration of 400 µg of inhaled albuterol underwent incremental symptom-limited cardiopulmonary exercise testing (CPET). The patients returned for two more experimental visits (3-7 days apart), during which they randomly received placebo or 0.5 mL of fenoterol hydrobromide (0.5% Berotec^(r)^; Boehringer Ingelheim do Brasil, São Paulo, Brazil) with 2 mL of ipratropium bromide (0.025% Atrovent^(r)^; Boehringer Ingelheim do Brasil) diluted in 5 mL of saline for nebulization. Within 30 min after nebulization, spirometry was performed, being followed by submaximal CPET at constant speed (i.e., at 70-80% of the maximum speed achieved during incremental CPET at the initial visit). During submaximal CPET at constant speed, serial measurements of IC were made every 2 min (from rest to peak exercise) in order to assess operating lung volumes during exercise. The study design is shown in Figure 1.

All spirometric tests were performed with a Koko PFT^(r)^ spirometer (PDS Instrumentation, Inc., Louisville, CO, USA). The variables measured were FVC, FEV_1_, FEV_1_/FVC, and IC. Maximal voluntary ventilation was estimated by multiplying FEV_1_ by 37.5.^(^ ^13^ ^)^ Participants completed at least three slow, forced expiratory maneuvers, considered acceptable and reproducible.

CPET was performed with the patients connected to a Vmax 229c^TM^ system (Vyasis, Yorba Linda, CA, USA) via a face mask and walking on an ATL treadmill (Inbrasport, Porto Alegre, Brazil). During incremental CPET, after 2 min at a constant speed of 1.6 km/h without inclination, the speed was increased every 1 min by 0.3 km/h, 0.5 km/h, or 0.8 km/h depending on the functional capacity of the patient, as determined by the examiner prior to the test. During the tests, the patients were instructed to hold the side bars only when needed (dizziness and loss of balance, among others). During submaximal CPET at constant speed, after a 2-min warm-up phase, the work rate was suddenly increased to a speed corresponding to 70-80% of the maximum speed achieved during incremental CPET, and the patients were encouraged to walk until they reached their limit of tolerance (Tlim, s). At the end of the initial phase and every 2 min during the tests, the patients were asked about the intensity of dyspnea and leg fatigue, by means of the modified Borg scale.^(^ ^14^ ^)^

The following variables were measured (breath by breath) and expressed as mean 15-s time: oxygen consumption, in mL/min, under standard temperature, pressure dry conditions; minute ventilation, in L/min, under body temperature, pressure saturated conditions; tidal volume, in L; and RR, in breaths/min. The R-R interval on a 12-lead electrocardiogram was used in order to determine HR (in bpm), and pulse oximetry with an Onyx 9500^TM^ pulse oximeter (Nonin, Plymouth, MN, USA) was used in order to estimate SpO_2_. We evaluated the dynamic changes in operating lung volumes by serial measurements of IC, assuming that TLC remained constant during exercise.^(^ ^9^ ^)^ During submaximal CPET at constant speed, two IC maneuvers were performed at rest, at the end of the initial period and every 2 min after the beginning of the constant speed test, in order to obtain reproducible values (< 10% difference in relation to the highest value, at each stage). In one of the visits after the administration of placebo (the second or third visit, depending on the randomization), the patients in whom IC at peak exercise was reduced in comparison with IC at rest were included in the DH+ group.^(^ ^15^ ^)^ A standardized time point near the end of the test marked "isotime", which was defined as the longest exercise duration common to the two submaximal cardiopulmonary exercise tests performed at constant speed by a given individual.

The data are presented as mean and standard deviation for variables with normal distribution and as median (interquartile range) for those with non-normal distribution. Possible differences between groups were analyzed by unpaired t-test, whereas differences between placebo and bronchodilator use were analyzed by paired t-test. Categorical variables were compared by means of Fisher's exact test. Changes in variables after placebo or bronchodilator use and the interaction depending on the presence or absence of DH during exercise were analyzed with the general linear model and multivariate repeated measures ANOVA. The Statistical Package for the Social Sciences, version 18.0 (SPSS Inc., Chicago, IL, USA), was used. The level of statistical significance was set at 5% for all tests (p < 0.05).

## Results

Of the 30 patients studied, 21 (70%) had DH during submaximal CPET at constant speed after placebo administration (the DH+ group) and 9 did not (the DH- group). There were no statistically significant differences between the DH+ group and the DH- group regarding age (67.9 ± 8.4 years vs. 66.1 ± 8.3 years), body mass index (26.6 ± 5.1 kg/m^2^ vs. 23.9 ± 4.4 kg/m^2)^, and maximal exercise capacity, which was determined by measuring oxygen consumption at peak exercise (1,400 ± 382 vs. 1,519 ± 243 mL/min).

After placebo administration, the proportion of patients with FEV_1_ < 50% of predicted was higher in the DH+ group (18/21; 86%) than in the DH- group (4/9; 44%; p = 0.016; Table 1). Surprisingly, however, resting IC tended to be higher in the DH+ group. All of the patients in the DH+ group had resting IC > 40% of predicted, as did 6 (67%) of the 9 patients in the DH- group (p = 0.02). The perception of dyspnea and leg fatigue during exercise was higher in the DH+ group than in the DH- group, whereas Tlim was lower in the former than in the latter (Table 1).

**Table 1:** Measurements taken before, during, and after constant-load exercise performed after placebo or bronchodilator use in the groups of patients with and without dynamic hyperinflation during exercise.a

Variables	Groups				
	DH+			DH-	
	(n = 21)			(n = 9)	
	PL	BD		PL	BD
Spirometry					
FEV_1_, L	1.01 ± 0.26	1.21 ± 0.36		1.32 ± 0.41*	1.55 ± 0.45*
FEV_1_, % of predicted	39 ± 11	46 ± 13		49 ± 16*	57 ± 15*
FVC, L	2.18 ± 0.46	2.52 ± 0.59		2.44 ± 0.43	2.77 ± 0.52
FVC, % of predicted	63 ± 11	72 ± 13		70 ± 18	80 ± 19
Resting IC, L	1.83 ± 0.57	1.89 ± 0.52		1.47 ± 0.32*	1.85 ± 0.44**^,^***
Isotime of exercise					
IC, L	1.50 ± 0.45	1.70 ± 0.51		1.61 ± 0.28	1.78 ± 0.28**
Δ IC isotime-rest, L	-0.32 ± 0.22	-0.19 ± 0.18**		0.14 ± 0.23*	-0.06 ± 0.26**^,^***
Dyspnea^b^	9.0 (7.0-10)	4.5 (2.0-10)**		4.0 (2.0-7.0)*	3.0 (1.0-7.0)***
Δ dyspnea BD-PL^b^		-3.5 (-6.0 to -1.0)			-1.0 (-3.0 to -4.0)***
Leg fatigue^b^	7 (3-10)	5 (2-10)		5 (3-8)*	5 (1-7)
Δ leg fatigue BD-PL^b^		-1.5 (-7.0 to -5.0)			-2.0 (-3.0 to -4.0)
End of exercise					
Tlim, s	423 ± 170	542 ± 258**		654 ± 255*	783 ± 261*^,^**
Dyspnea^b^	9.0 (7.0-10)	7.5 (1.0-10)		4.5 (2.0-7.0)*	4.5 (1.0-9.0)
Leg fatigue^b^	7.0 (7.0-10)	7.0 (1.0-10)		5.0 (3.0-8.0)*	5.5 (0.0-9.0)
SpO_2_, %	91 ± 6	92 ± 5		87 ± 8	89 ± 9

PL : placebo

BD : bronchodilator

IC : inspiratory capacity

**Figure 2:**
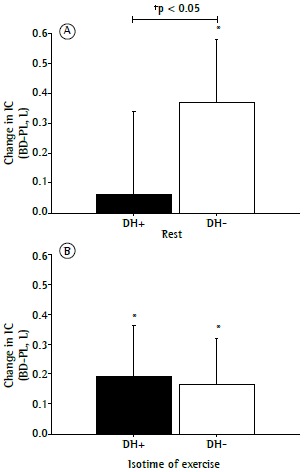
Change in inspiratory capacity bronchodilator-placebo (BD-PL) at rest (in A) and at isotime of exercise at constant speed (in B) in the groups of patients with dynamic hyperinflation (DH+) and without dynamic hyperinflation (DH-). *p < 0.05; intragroup BD-PL difference. †Intergroup BD-PL difference.

**Figure 3:**
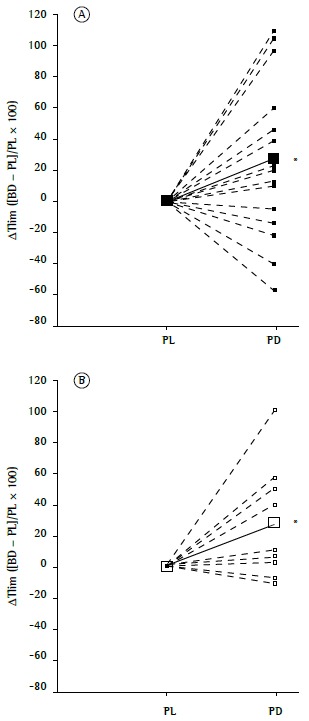
Bronchodilator/placebo (BD-PL) change in exercise tolerance (Tlim) in the patients (dashed lines) with dynamic hyperinflation (in A) and without dynamic hyperinflation (in B). The solid lines represent the group means. *p < 0.05; intragroup difference.

Bronchodilator use resulted in equivalent gains in FEV_1_ in the DH+ and DH- groups, with significant increases in flow, which were determined in accordance with the Brazilian Thoracic Association criteria (7/19; 37% vs. 5/9; 56%).^(^ ^16^ ^)^ However, the variations in resting IC after bronchodilator use were lower in the DH+ group than in the DH- group (Figure 2A). All of the patients in the DH- group showed an increase in resting IC, as did 9 (43%) of the 21 patients in the DH+ group (p < 0.01), resting IC values being therefore equalized (Table 1). Our analysis of operating lung volumes after bronchodilator use showed that IC gains at peak exercise were similar between the two groups (Figure 2B). Although the reduction in dyspnea was greater in the DH+ group than in the DH- group, both groups showed similar improvements in Tlim with the use of placebo (median [interquartile range]: 22% [3-60%] vs. 10% [3-53%]; p > 0.05; Figure 3).

## Discussion

The main finding of the present study was a significant increase in Tlim after bronchodilator use, regardless of the previous pattern of DH during exercise (Figure 3). The increase in resting IC after bronchodilator use-reflecting increased static hyperinflation-was associated with increased Tlim in the DH- group. Bronchodilator use improved exercise performance in patients who showed no improvement in resting IC, although only in those who had DH. Therefore, bronchodilator use can improve exercise tolerance in COPD patients by reducing static hyperinflation at rest and by reducing the rate of hyperinflation during exercise.

Given that DH plays a central role in limiting exercise in COPD patients,^(^ ^1^ ^-^ ^3^ ^)^ a reduction in DH after bronchodilator use^(^ ^5^ ^)^ (as evidenced by a significant increase in IC at isotime; Figure 2B) was expected to result in increased Tlim in the DH+ patients. Given that reduced DH has consistently been associated with increased endurance time,^(^ ^6^ ^,^ ^17^ ^-^ ^19^ ^)^ the DH+ patients were expected to have a more favorable pathophysiological substrate for bronchodilator activity and show significantly greater increases in Tlim when compared with the DH- patients. However, both groups showed similar improvements in Tlim after bronchodilator use (Figure 3). Although from a conceptual standpoint the DH- patients did not develop DH, the significant increase in resting IC (Figure 2A) seems to have represented an important mechanism to explain improved exercise performance.

Resting IC has been identified as an important modulator of ventilatory capacity, breathing pattern, dyspnea on exertion,^(^ ^11^ ^)^ and Tlim^(^ ^10^ ^,^ ^17^ ^)^ in patients with COPD. This means that static lung volume measurements provide an estimate of the inspiratory reserve volume available for exercise, delaying a critical limitation in tidal volume expansion.^(^ ^20^ ^)^ Therefore, the development of ventilatory constraint seems to be the primary component influencing the pattern of respiratory response to exercise in patients with COPD. This important mechanic event during exercise marks the beginning of the progressive disparity between respiratory muscle effort (together with central nervous stimulation) and thoracic movement (neuromechanical dissociation), resulting in intolerable levels of dyspnea and in exercise termination.^(^ ^20^ ^,^ ^21^ ^)^ Therefore, a low IC at rest (reflecting static lung hyperinflation) and a critical reduction in IC during exercise (DH) can, in isolation or in combination, limit the ability to increase ventilation or reach a critical inspiratory reserve volume that, limited superiorly by TLC, does not allow a further increase in tidal volume.^(^ ^11^ ^)^

Previous studies (including a total of 100 patients) have shown that the pattern of DH influences exercise capacity.^(^ ^22^ ^-^ ^25^ ^)^ In contrast, Guenette et al.^(^ ^26^ ^)^ recently analyzed a total of 130 COPD patients (whose FEV1 values were similar to those observed in previous studies, i.e., ~ 40-50% of predicted) and reported that the presence or absence of DH during exercise had no influence on the intensity of dyspnea or on exercise tolerance during high-intensity exercise. On the contrary, critical restriction of tidal volume expansion was shown to be the primary mechanism associated with those outcomes, independently of the presence of DH. In addition, the reduction in dyspnea after bronchodilator therapy, hyperoxia, and physical training has been shown to occur independently of the reduction in the rate of DH.^(^ ^27^ ^-^ ^29^ ^)^ Therefore, it is likely that other mechanical effects (including an absolute reduction in operating lung volumes with a delay in reaching a critical restriction of tidal volume expansion) occurring after these interventions are more important in explaining the improvement in dyspnea and exercise tolerance than is the small or inconsistent reduction in the rate of development of DH. It is of note that the patients in the DH+ group had higher dyspnea scores at isotime than did those in the DH- group. This finding is consistent with the concept that the magnitude of dyspnea is related to ventilation at increased operating volumes (reduced IC) and the resulting neuromechanical uncoupling.^(^ ^20^ ^,^ ^21^ ^)^

The mechanism whereby the DH- patients in the present study were able to achieve increased IC during exercise (in comparison with reduced IC at rest) after placebo administration remains unexplained. Similar results were obtained in a previous study,^(^ ^30^ ^)^ in which it was speculated that the abovementioned finding was due to lower expiratory airflow limitation in less severely ill patients, with a respiratory pattern of abdominal muscle recruitment during exercise and, consequently, reduced operating lung volumes. However, unlike the patients in the present study, the patients in that study showed lower Tlim after bronchodilator use than did those who were more severely ill and who had hyperinflation.

The main limitations of the present study include the fact that we evaluated a convenience sample, having recruited patients during a predetermined period (possibly resulting in insufficient statistical power to make certain comparisons), and the fact that we did not measure TLC. This means that the variations in lung volumes were estimated exclusively by IC, rather than by EELV (i.e., TLC/IC). Although this limitation did not allow us to evaluate, in an adequate manner, possible differences in the baseline degree of positioning of operating lung volumes, this was minimized by the crossover design of the study, in which the same individuals were compared after two different interventions. In addition, we did not study patients with severe hypoxemia (resting SpO2 < 90%), in whom the hypoxic drive can modulate the kinetics of DH development and the bronchodilator response. Therefore, our findings should not be extrapolated to such patients.

In conclusion, the heterogeneity of the pattern of development of DH during exercise does not seem to modulate the ability of patients with moderate to severe COPD to improve their exercise capacity after inhaled bronchodilator use. Therefore, increased exercise tolerance in DH- patients seems to be related to a bronchodilator-induced reduction in resting "static" lung hyperinflation. However, patients showing no deflation at rest could still benefit from bronchodilator use, provided that there is a decrease in the rate of development of DH during exercise. Clinically, these data demonstrate that measurements of IC at rest and during exercise are complementary in the evaluation of the mechanisms underlying the beneficial effects of bronchodilators in this population of patients.
