# Supplementary material for: Can bronchodilators improve exercise tolerance in COPD patients without dynamic hyperinflation?
Source: J Bras Pneumol. 2014 Mar-Apr;40(2):111–8. doi: 10.1590/S1806-37132014000200003 (PMC4083636; doi:10.1590/S1806-37132014000200003)
Supplement: Translation

# Translation: Os broncodilatadores podem melhorar a tolerância ao exercício na ausência de hiperinsuflação dinâmica em pacientes com DPOC?^*^

**Authors:** Maria Enedina Aquino Scuarcialupi, Danilo Cortozi Berton, Priscila Kessar Cordoni, Selma Denis Squassoni, Elie Fiss, José Alberto Neder

**Affiliations:** Faculdade de Ciências Médicas da Paraíba, João Pess oa (PB) Brasil; Faculdade de Medicina, Universidade Federal do Rio Grande do Sul; e Pneumologista. Hospital de Clínicas de Porto Alegre, Porto Alegre (RS) Brasil; Disciplina de Pneumologia, Faculdade de Medicina do ABC, Santo An dré (SP) Brasil; Setor de Reabilitação Pulmonar, Disciplina de Pneumologia, Faculdade de Medicina do ABC, Santo André (SP) Brasil; Disciplina de Pneumologia, Faculdade de Medicina do ABC, Santo André (SP) Brasil

**Keywords:** Doença pulmonar obstrutiva crônica, Broncodilatadores, Teste de esforço, Tolerância ao exercício, Capacidade inspiratória

## Abstract

**OBJETIVO:**

Investigar os efeitos moduladores da hiperinsuflação dinâmica (HD), definida pela redução da capacidade inspiratória (CI), na tolerância ao exercício após broncodilatador em pacientes com DPOC.

**MÉTODOS:**

Estudo experimental e randomizado com 30 pacientes com DPOC sem hipoxemia grave. Na visita inicial, os pacientes realizaram avaliação clínica, espirometria e teste de exercício cardiopulmonar (TECP) incremental. Em duas visitas subsequentes, os pacientes foram randomizados para receber uma combinação de fenoterol/ipratrópio ou placebo e, em seguida, realizaram espirometria e TECP com velocidade constante até o limite da tolerância (Tlim). Os pacientes com ΔCI(pico-repouso) < 0 foram considerados com HD (HD+).

**RESULTADOS:**

Nesta amostra, 21 pacientes (70%) apresentaram HD. Os pacientes HD+ apresentaram maior obstrução ao fluxo aéreo e menor Tlim do que os pacientes sem HD (HD-). Apesar de ganhos equivalentes de VEF_1_ após broncodilatador, o grupo HD- apresentou maior ΔCI(broncodilatador-placebo) em repouso em relação ao grupo HD+ (p < 0,05). Entretanto, isso não ocorreu com a ΔCI no pico do exercício entre os grupos HD+ e HD- (0,19 ± 0,17 L vs. 0,17 ± 0,15 L; p > 0,05). Similarmente, ambos os grupos apresentaram melhoras equivalentes do Tlim após broncodilatador (mediana [intervalo interquartílico]: 22% [3-60%] e 10% [3-53%]; p > 0,05).

**CONCLUSÕES:**

A melhora da CI em repouso após broncodilatador associou-se com ganho de tolerância ao esforço mesmo nos pacientes com DPOC que não apresentem HD. Por outro lado, pacientes sem melhora da CI em repouso ainda podem obter beneficio funcional com o broncodilatador desde que apresentem HD no exercício.

## Introdução

A hiperinsuflação pulmonar é um mecanismo crucial da dispneia ao exercício na DPOC.^(^ ^1^ ^-^ ^3^ ^)^ A terapia com broncodilatadores pode reduzir os volumes pulmonares estáticos e dinâmicos determinados durante o exercício, aumentando a tolerância ao exercício nesses pacientes.^(^ ^4^ ^,^ ^5^ ^)^

O conceito atual dos mecanismos pelos quais os broncodilatadores podem melhorar a tolerância ao exercício na DPOC enfatiza a capacidade de redução da taxa de aumento do volume pulmonar expiratório final (VPEF) com a progressão do exercício, isto é, redução da hiperinsuflação dinâmica (HD).^(^ ^1^ ^,^ ^6^ ^)^ Na prática, a HD pode ser estimada por medidas seriadas da capacidade inspiratória (CI),^(^ ^6^ ^-^ ^8^ ^)^ a qual vai refletir o VPEF, considerando que a CPT não muda consideravelmente com o exercício.^(^ ^9^ ^)^ Um mecanismo alternativo - ou complementar - da ação dos broncodilatadores seria o da redução dos volumes pulmonares operantes no repouso, ou seja, a desinsuflação pré-exercício.^(10,11) ^Nesse caso, os pacientes ainda poderiam obter benefícios com o broncodilatador mesmo na ausência de HD, já que haveria maior "reserva volumétrica" a ser consumida durante o esforço. Em qualquer circunstância, os pacientes poderiam alcançar, com o uso do broncodilatador, o mesmo VPEF no pico do exercício, mas por mecanismos distintos: menor taxa de HD ou menor hiperinsuflação estática.

Objetivamos, dessa forma, investigar se a administração de broncodilatador poderia causar uma melhora similar na capacidade de exercício, apesar de atuar predominantemente na hiperinsuflação estática ou dinâmica relacionada ao exercício, em pacientes com DPOC moderada a grave. A confirmação dessa premissa embasaria a noção de que a medida de hiperinsuflação pulmonar, tanto em repouso quanto aquela relacionada ao exercício, é complementar na avaliação dos benefícios dos broncodilatadores na tolerância ao exercício desses pacientes.

## Métodos

Foi estudada uma amostra de conveniência com 30 pacientes diagnosticados com DPOC, conforme os critérios da *Global Initiative for Chronic Obstructive Lung Disease*,^(^ ^12^ ^)^ que apresentavam VEF_1_ pós-broncodilatador < 70% do previsto, relação VEF_1_/CVF < 70%, história de tabagismo (> 20 anos-maço) e idade superior a 40 anos. Os pacientes foram recrutados do ambulatório de DPOC e do centro de reabilitação pulmonar da instituição. Foram considerados critérios de exclusão a presença de hipoxemia grave em repouso (SpO_2_ < 90%); comorbidades que pudessem contribuir para dispneia e limitação ao exercício; exacerbação da DPOC ou infecção respiratória no mês anterior; ou qualquer contraindicação para a realização de teste de exercício clínico. O projeto foi aprovado pelo comitê de ética em pesquisa da instituição. Todos os pacientes assinaram termo de consentimento livre e esclarecido.

Na visita inicial, após a realização de avaliação clínica e funcional com espirometria antes e após a administração de 400 µg de salbutamol por via inalatória, se mantida a elegibilidade dos pacientes, era realizado o teste de exercício cardiopulmonar incremental limitado por sintomas (TECPinc). Posteriormente, os pacientes retornavam para mais duas visitas experimentais, separadas entre si por 3-7 dias, durante as quais recebiam, de forma aleatória, placebo ou 0,5 mL de bromidrato de fenoterol (Berotec^(r)^ 0,5%; Boehringer Ingelheim do Brasil, São Paulo, Brasil) associado a 2 mL de brometo de ipratrópio (Atrovent^(r)^ 0,025%; Boehringer Ingelheim do Brasil) em solução para nebulização diluída em 5 mL de solução fisiológica. Após 30 min da administração da nebulização, era realizada espirometria e, em seguida, TECP submáximo de alta intensidade com velocidade constante (TECPct) a 70-80% da velocidade máxima obtida no TECPinc da visita inicial. Durante a realização do TECPct, medidas seriadas da CI foram feitas a cada 2 min (do repouso ao pico) visando avaliar os volumes pulmonares operantes durante o exercício. O delineamento do estudo está representado na Figura 1.

**Figura 1:** Delineamento do estudo. BD: broncodilatador; TECP: teste de exercício cardiopulmonar: e R: randomização.

O aparelho Koko PFT^(r)^ (PDS Instrumentation, Inc., Louisville, CO, EUA) foi usado para a realização das espirometrias. As variáveis mensuradas foram CVF, VEF_1_, relação VEF_1_/CVF e CI. A ventilação voluntária máxima foi estimada pelo cálculo VEF_1_ × 37,5.^(^ ^13^ ^)^ Os indivíduos completaram pelo menos três manobras, forçadas e lentas, aceitáveis e reprodutíveis.

Os TECP foram realizados com os pacientes conectados, via máscara facial, a um sistema Vmax 229c^TM^ (Vyasis, Yorba Linda, CA, EUA), exercitando-se em uma esteira rolante modelo ATL (Inbrasport, Porto Alegre, Brasil). No TECPinc, após 2 min numa velocidade padronizada de 1,6 km/h, sem inclinação, a velocidade era aumentada, a cada minuto, em 0,3, 0,5 ou 0,8 km/h, de acordo com a capacidade funcional do paciente, que foi julgada previamente ao teste pelo examinador. Durante os testes, os pacientes foram orientados a se apoiarem levemente nas barras laterais de suporte apenas em caso de necessidade (tonturas, perda de equilíbrio, etc.). No TECPct, após a fase de aquecimento de 2 min, a taxa de trabalho aumentava subitamente para uma velocidade que correspondia a 70-80% da velocidade máxima atingida no TECPinc, sendo o paciente estimulado a caminhar até o limite da tolerância (Tlim, s). No final da fase inicial e a cada 2 min durante os testes, os pacientes foram questionados quanto à intensidade da dispneia e fadiga nos membros inferiores usando a escala de Borg modificada.^(^ ^14^ ^)^

As seguintes variáveis foram medidas, respiração a respiração e expressas como média em 15 s: consumo de oxigênio, em mL/min, em condições *standard temperature, pressure dry* (STPD); ventilação minuto, em L/min, em condições *body temperature, pressure saturated*; volume corrente, em L; e FR, em ciclos/min. A FC, em bpm, foi determinada usando o intervalo R-R de um eletrocardiograma de 12 derivações, e a SpO_2_ foi estimada por oximetria de pulso (Onyx 9500^TM^; Nonin, Plymouth, MN, EUA). As modificações dinâmicas nos volumes pulmonares operantes foram avaliadas com medidas seriadas da CI, assumindo-se que a CPT permanece constante durante o exercício.^(^ ^9^ ^)^ Durante o TECPct, foram realizadas duas manobras de CI em repouso, ao final do período inicial e a cada 2 min após o início da velocidade constante, objetivando-se obter valores reprodutíveis (diferença menor que 10% do maior valor, em cada estágio). Em uma das visitas após a administração de placebo (a segunda ou a terceira, conforme a randomização), os pacientes que tiveram qualquer redução da CI no pico do exercício em relação à CI em repouso foram incluídos no grupo HD+.^(^ ^15^ ^)^ Um tempo padronizado próximo ao término do teste (isotempo) foi definido como o maior tempo de exercício comum atingido nos dois TECPct realizados por um determinado indivíduo.

Os dados são apresentados como média e desvio-padrão para as variáveis com distribuição simétrica e como mediana (intervalo interquartílico) para aquelas com distribuição assimétrica. Possíveis diferenças entre os grupos foram investigadas pelo teste t não pareado, enquanto diferenças comparando a utilização de placebo e de broncodilatador pelo teste t pareado. A comparação das variáveis categóricas foi feita com o teste exato de Fisher. As análises da alteração das variáveis após o uso de placebo ou de broncodilatador e da interação conforme o padrão de HD ao exercício (presente ou ausente) foram feitas usando o modelo linear geral e ANOVA multivariada para medidas repetidas. O pacote estatístico utilizado foi o *Statistical Package for the Social Sciences*, versão 18.0 (SPSS Inc., Chicago, IL, EUA). O nível de significância estatística foi definido em 5% para todos os testes (p < 0,05).

## Resultados

Do total de 30 pacientes, 21 (70%) apresentaram HD durante o TECPct após o uso de placebo (grupo HD+). Não houve diferenças estatisticamente significantes entre os grupos HD+ e sem HD (HD-) em relação a idade (67,9 ± 8,4 anos vs. 66,1 ± 8,3 anos), índice de massa corpórea (26,6 ± 5,1 kg/m^2 ^vs. 23,9 ± 4,4 kg/m^2)^ e capacidade máxima de exercício, expressa pelo consumo de oxigênio no pico do exercício (1.400 ± 382 vs. 1.519 ± 243 mL/min).

Após a administração de placebo, uma maior proporção de pacientes (18/21; 86%) do grupo HD+ apresentaram VEF_1_ < 50% do previsto do que os do grupo HD- (4/9; 44%; p = 0,016; Tabela 1). Surpreendentemente, entretanto, os pacientes HD+ tenderam a apresentar maior CI em repouso. Todos pacientes HD+ tiveram CI em repouso > 40% do previsto, enquanto 6/9 (67%) dos pacientes HD- apresentaram esse resultado (p = 0,02). Os pacientes HD+ tiveram maior percepção de dispneia e de fadiga nos membros inferiores no exercício, bem como menor Tlim em relação ao grupo HD- (Tabela 1).

**Tabela 1:** Medidas antes, durante e após exercício de carga constante com o uso de placebo ou de broncodilatador nos grupos de pacientes com e sem hiperinsuflação dinâmica ao esforço.a

Variáveis	Grupos			
	HD+		HD-	
	(n = 21)		(n = 9)	
	PL	BD	PL	BD
Espirometria				
VEF_1, _L	1,01 ± 0,26	1,21 ± 0,36	1,32 ± 0,41*	1,55 ± 0,45*
VEF_1, _% previsto	39 ± 11	46 ± 13	49 ± 16*	57 ± 15*
CVF, L	2,18 ± 0,46	2,52 ± 0,59	2,44 ± 0,43	2,77 ± 0,52
CVF, % previsto	63 ± 11	72 ± 13	70 ± 18	80 ± 19
CI repouso, L	1,83 ± 0,57	1,89 ± 0,52	1,47 ± 0,32*	1,85 ± 0,44**^,^***
Exercício, isotempo				
CI, L	1,50 ± 0,45	1,70 ± 0,51	1,61 ± 0,28	1,78 ± 0,28**
Δ CI isotempo-repouso, L	-0,32 ± 0,22	-0,19 ± 0,18**	0,14 ± 0,23*	-0,06 ± 0,26**^,^***
Dispneia^b^	9,0 (7,0-10)	4,5 (2,0-10)**	4,0 (2,0-7,0)*	3,0 (1,0-7,0)***
Δ Dispneia BD-PL^b^		-3,5 (-6,0 a -1,0)		-1,0 (-3,0 a -4,0)***
Fadiga MMII^b^	7 (3-10)	5 (2-10)	5 (3-8)*	5 (1-7)
Δ Fadiga em MMII BD-PL^b^		-1,5 (-7,0 a -5,0)		-2,0 (-3,0 a -4,0)
Exercício, final				
Tlim, s	423 ± 170	542 ± 258**	654 ± 255*	783 ± 261*^,^**
Dispneia^b^	9,0 (7,0-10)	7,5 (1,0-10)	4,5 (2,0-7,0)*	4,5 (1,0-9,0)
Fadiga MMII^b^	7,0 (7,0-10)	7,0 (1,0-10)	5,0 (3,0-8,0)*	5,5 (0,0-9,0)
SpO_2_, %	91 ± 6	92 ± 5	87 ± 8	89 ± 9

PL : placebo

BD : broncodilatador

CI : capacidade inspiratória

MMII : membros inferiores

Tlim : tempo de tolerância ao exercício

a Valores apresentados em média ± dp, exceto onde indicado

b Valores apresentados em mediana (intervalo interquartílico). Escala modificada de Borg

* p < 0,05: variação intergrupos num dado momento (PL ou BD)

** p < 0,05: variação intrassujeitos (pré vs. pós-BD)

*** p < 0,05 variação intergrupos (pré vs. pós-BD).

A administração de broncodilatador determinou ganhos equivalentes de VEF_1_ entre os pacientes dos grupos HD+ e HD-, que tiveram aumentos significativos no fluxo conforme os critérios da Sociedade Brasileira de Pneumologia e Tisiologia (7/19; 37% vs. 5/9; 56%).^(^ ^16^ ^)^ Entretanto, o grupo HD+ apresentou, após o uso do broncodilatador, menores variações da CI em repouso do que o grupo HD- (Figura 2A). Todos os pacientes HD- apresentaram um aumento da CI em repouso comparado a 9/21 (43%) dos pacientes HD+ (p < 0,01) e, dessa forma, os valores de CI de repouso foram equalizados (Tabela 1). A análise dos volumes operacionais após o uso de broncodilatador demonstrou ganhos similares de CI no pico de exercício em ambos os grupos (Figura 2B). Apesar de maior redução da dispneia nos pacientes HD+, os grupos HD+ e HD- apresentaram melhoras equivalentes da Tlim em relação ao uso de placebo (mediana [intervalo interquartílico]: 22% [3-60%] vs. 10% [3-53%], respectivamente; p > 0,05; Figura 3).

**Figura 2:** Variação da capacidade inspiratória broncodilatador-placebo (BD-PL) no repouso (em A) e no isotempo do exercício de velocidade constante (em B) nos grupos com hiperinsuflação dinâmica (HD+) e sem hiperinsuflação dinâmica (HD-). *p < 0,05; diferença BD-PL intragrupo. †Diferença intergrupos BD-PL.

**Figura 3:** Variação broncodilatador-placebo (BD-PL) do tempo de tolerância ao exercício (Tlim) nos pacientes (linhas tracejadas) com hiperinsuflação dinâmica (em A) e sem hiperinsuflação dinâmica (em B). As linhas contínuas representam as médias dos grupos. *p < 0,05; diferença intragrupo.

## Discussão

O principal achado do presente estudo foi a demonstração do aumento significativo do Tlim após o uso de broncodilatador, independentemente do padrão prévio de desenvolvimento de HD no exercício (Figura 3). Observou-se que o incremento da CI em repouso após o uso de broncodilatador - refletindo melhora da hiperinsuflação estática - foi associado com o aumento da Tlim nos pacientes HD-. Por sua vez, o fato de alguns pacientes não apresentarem um aumento da CI em repouso não impossibilitou que a administração de broncodilatador melhorasse o desempenho no exercício, desde que os mesmos apresentassem HD. Portanto, pacientes com DPOC podem apresentar melhora da tolerância ao exercício após o uso de broncodilatador tanto pela diminuição da hiperinsuflação estática em repouso quanto pela redução da taxa de hiperinsuflação no exercício.

Considerando a presença de HD como fator central da limitação ao exercício na DPOC,^(^ ^1^ ^-^ ^3^ ^)^ seria previsível que a atenuação do seu desenvolvimento nos pacientes HD+ com o uso de broncodilatador^(^ ^5^ ^)^ (conforme demonstrado pelo aumento significativo da CI no isotempo; Figura 2B) resultasse em aumento do Tlim. Como a redução da HD tem sido reiteradamente associada com o aumento do tempo de *endurance*,^(^ ^6^ ^,^ ^17^ ^-^ ^19^ ^)^ era de se supor que os pacientes HD+ teriam um substrato fisiopatológico mais favorável para a atuação broncodilatadora e apresentariam aumentos significativamente maiores do Tlim comparado aos pacientes HD-. Entretanto, aumentos similares do Tlim após o uso de broncodilatador foram observados entre os grupos (Figura 3). Embora conceitualmente o grupo HD- não tenha desenvolvido HD, o aumento significativo da CI em repouso (Figura 2A) parece ter representado um mecanismo importante para explicar o melhor desempenho no exercício.

A CI de repouso tem sido identificada como importante moduladora de capacidade ventilatória, padrão respiratório, dispneia ao exercício^(^ ^11^ ^)^ e Tlim^(^ ^10^ ^,^ ^17^ ^)^ na DPOC. Isso significa que as medidas dos volumes estáticos fornecem uma estimativa do volume de reserva inspiratório disponível para o exercício, retardando o aparecimento da limitação crítica na expansão do volume corrente.^(^ ^20^ ^)^ Assim, o desenvolvimento de limitação (*constraint*) ventilatória parece ser o componente primário que influencia o padrão de resposta respiratória ao exercício em pacientes com DPOC. Esse importante evento mecânico durante o exercício marca o início da disparidade progressiva entre o esforço muscular respiratório despendido (junto com o estímulo nervoso central) e o movimento torácico realmente efetuado (dissociação neuromecânica), que resulta em níveis intoleráveis de dispneia e interrupção do exercício.^(^ ^20^ ^,^ ^21^ ^)^ Portanto, uma baixa CI em repouso (refletindo hiperinsuflação pulmonar estática) ou a sua redução crítica no exercício (HD) pode, isolada ou complementarmente, limitar a capacidade de aumentar a ventilação: atingir um volume crítico de reserva inspiratória, que, limitado superiormente pela CPT, não permite um aumento adicional do volume corrente.^(^ ^11^ ^)^

Trabalhos prévios (estudando um total de 100 pacientes) demonstraram que o padrão de HD influencia a capacidade de exercício.^(^ ^22^ ^-^ ^25^ ^)^ Por outro lado, mais recentemente, Guenette et al.,^(^ ^26^ ^)^ analisando um total de 130 pacientes com DPOC (na mesma faixa aproximada de gravidade do VEF_1_ dos estudos anteriores, ou seja, ~ 40-50% do previsto), descreveram que a presença ou a ausência de HD no exercício não influenciou a intensidade da dispneia ou a tolerância durante exercício de alta intensidade. Pelo contrário, foi demonstrado que a limitação crítica na expansão do volume corrente foi o principal mecanismo associado com esses desfechos, independentemente da presença de HD. Além disso, a redução da dispneia após terapia broncodilatadora, hiperóxia e treinamento físico tem mostrado ocorrer independentemente da redução na taxa de HD.^(27-29) ^Portanto, é provável que outros efeitos mecânicos (como a redução absoluta dos volumes pulmonares operantes com um atraso no alcance da limitação crítica na expansão do volume corrente) após essas intervenções sejam mais importantes em explicar a melhora da dispneia e da tolerância ao exercício do que a pequena ou inconsistente redução na taxa de desenvolvimento de HD. Deve-se observar que os pacientes do grupo HD+ reportaram maiores escores de dispneia no isotempo comparativamente ao grupo HD-. Tal achado é compatível com o conceito de que a magnitude da dispneia está relacionada à ventilação em volumes operantes mais altos (menor CI) e o resultante desacoplamento neuromecânico.^(^ ^20^ ^,^ ^21^ ^)^

O mecanismo pelo qual os pacientes HD- do presente estudo conseguiram, mesmo partindo de valores menores de CI em repouso, ter um aumento em sua CI durante o exercício com o uso de placebo permanece sem explicação. Resultados parecidos foram encontrados em um estudo prévio,^(^ ^30^ ^)^ no qual foi especulado que a menor limitação expiratória em pacientes menos graves, com padrão respiratório de recrutamento da musculatura abdominal durante o exercício e consequente redução dos volumes pulmonares operantes, poderia justificar tal achado. Entretanto, diferentemente dos nossos resultados, os pacientes daquele estudo apresentaram, após o uso de broncodilatador, menor Tlim quando comparados aos pacientes mais graves e que apresentavam hiperinsuflação.

As principais limitações do presente estudo são as seguintes: foi avaliada uma amostra de conveniência, recrutando pacientes em um período pré-determinado, o que pode ter resultado em um poder estatístico insuficiente para algumas comparações, e não houve mensuração da CPT. Assim, as variações dos volumes foram estimadas somente a partir da CI e não do verdadeiro VPEF (isto é, CPT - CI). Embora tal limitação não tenha permitido a avaliação adequada de possíveis diferenças no grau basal de posicionamento dos volumes pulmonares operantes, isso foi minimizado pelo delineamento cruzado do estudo, comparando os mesmos indivíduos após as diferentes intervenções. Deve-se ressaltar, ainda, que não foram estudados pacientes hipoxêmicos graves (SpO_2 _< 90% em repouso), nos quais o comando hipóxico poderia modular a cinética de desenvolvimento de HD e a resposta ao broncodilatador. Portanto, nossos achados não podem ser extrapolados para esses pacientes.

Em conclusão, a heterogeneidade no padrão de desenvolvimento de HD ao exercício não parece modular a habilidade de pacientes com DPOC moderada a grave de melhorar a capacidade de exercício após o uso de broncodilatador inalatório. Dessa forma, o aumento da tolerância ao exercício em pacientes HD- parece se relacionar com a redução da hiperinsuflação "estática" em repouso induzida pelo broncodilatador. Por outro lado, tal tratamento pode também beneficiar pacientes que não apresentam desinsuflação em repouso, desde que haja uma diminuição da taxa de desenvolvimento de HD no exercício. Clinicamente, esses dados demonstram que medidas de CI em repouso e no exercício são complementares para a avaliação dos mecanismos subjacentes aos efeitos benéficos dos broncodilatadores nessa população de pacientes.
